# Ciliated hepatic foregut cyst: a case report and review of literature on a rare hepatic cystic lesion

**DOI:** 10.1097/MS9.0000000000004098

**Published:** 2025-10-30

**Authors:** Abdullah Saleh AlQattan, Mohammed Abdulmohsen AlSharit, Abdullah A. AlQatari, Lujain Binkhamis, Hassan Al Zaharani, Nabeel Mansi

**Affiliations:** a Department of Surgery, King Fahad Specialist Hospital, Dammam, Saudi Arabia; b Department of Surgery, King Faisal Specialist Hospital and Research Center, Riyadh, Saudi Arabia; c Department of Surgery, King Fahad Hospital of the University, Khobar, Saudi Arabia; d Department of Radiology, King Fahad Specialist Hospital, Dammam, Saudi Arabia

**Keywords:** ciliated hepatic foregut cyst, liver, liver resection, squamous cell carcinoma

## Abstract

**Introduction:**

Ciliated hepatic foregut cyst (CHFC) is one of the rarely reported cystic lesions of the liver, with approximately 200 cases reported in the literature. Although most patients with CHFC are asymptomatic and diagnosed incidentally, they should be investigated thoroughly to rule out other possible differential diagnoses, as CHFCs carry a risk of malignant transformation into squamous carcinoma.

**Case presentation:**

A 19-year-old female was found to have a liver lesion during laparoscopic appendectomy for acute appendicitis. Post-appendectomy liver imaging revealed a solitary 6.4 cm well-defined, multilocular cystic lesion centered in the left liver without features of malignancy. There was no communication with the biliary system; however, there was mild dilatation of the intrahepatic biliary radicles. The differential diagnoses included biliary cystadenoma, simple cyst, and cystic biliary hamartoma. Based on these findings and the absence of malignant features in the cyst, an open cholecystectomy and nonanatomical resection of segments 4/5 was performed. The patient had an uneventful postoperative recovery and was discharged in good condition. Histopathological examination revealed a CHFC, and the patient was followed up for 2 years without complications.

**Discussion:**

CHFC is a rare benign liver cyst with the potential for malignant transformation. Among 223 reported cases, 2.7% were malignant. Most lesions are found incidentally, typically in segment IV. Owing to the variable imaging features, a definitive diagnosis requires histological confirmation. Larger cysts or those with irregular walls pose a higher risk. Surgical excision, preferably laparoscopic, is the treatment of choice, with no reported recurrences. The role of surveillance for small, asymptomatic cysts remains controversial.

**Conclusion:**

Accurate preoperative diagnosis of CHFC is often challenging. Nevertheless, given its potential for malignant transformation, a high index of suspicion is warranted in such cases. When clinical and radiological features raise concerns, complete surgical excision remains the treatment of choice.

## Introduction

Ciliated hepatic foregut cysts (CHFCs) were first described in the literature by the German pathologist Nicolaus Friedreich in 1857. At the time, they were called “cystic lesions of the liver with foregut embryologic origin.” It was not until the late 1980s that they were designated as CHFCs by Wheeler and Edmonson^[1]^. About 223 CHFC cases have been published, with only 6 (2.7%) of all reported cases showing malignant transformation (Table 1)^[1,2]^. CHFC is an exceedingly rare and benign cystic lesion that develops from the embryonic remnant of the foregut epithelium^[3]^. Nevertheless, its rarity should not undermine the importance of accurate diagnosis. Accuracy is particularly important because cases of malignant transformation from CHFCs that exhibit a radiological resemblance to some malignant cystic lesions of the liver have been reported^[3,4]^. In this article, we report a case of CHFC and review the literature to summarize the current evidence guiding management. This case report has been reported in line with the SCARE checklist^[5]^.

**HIGHLIGHTS**

- Ciliated hepatic foregut cyst (CHFC) is a rare liver lesion with malignant potential, arising from embryonic foregut remnants.
- Surgical resection is a common treatment option.
- The clinical presentation of CHFC is variable; however, most reported cases, including ours, have been diagnosed incidentally. Although various radiological modalities can aid in identifying CHFC, imaging alone is insufficient for a definitive diagnosis due to the lesion’s diverse appearance. Given the rarity of this condition, further research is needed to enhance understanding and develop more precise management guidelines.

**Table 1:** All the reported cases of CHFC with malignant transformation

Author (year)	Age/Sex	Symptoms	Cyst size	Location	Treatment	Outcome
Vick *et al* (1999)^[6]^	51/M	Abdominal pain	12 cm	Right lobe	Resection	No recurrence (short follow-up)
Furlanetto *et al* (2002)^[7]^	21/M	Pain, weight loss	10 cm	Segments 5–6	Resection + chemotherapy	Death (metastasis)
A. S. de Lajarte-Tirouard *et al* (2002)^[8]^	40/F	Abdominal pain	13 cm	Segment 5	Resection	No recurrence
Zhang *et al* (2009)^[9]^	60/F	Abdominal fullness	7 cm	Segment 4	Resection	Alive, no recurrence
Wilson *et al* (2013)^[10]^	34/M	Asymptomatic	14 cm	Segments 4, 5, and 8	Resection + TACE + chemotherapy	Recurrence + metastasis
Itose *et al* (2020)^[11]^	50/F	Abdominal pain	4 cm	Segment 4	Resection	Recurrence (alive at 30 m)

## Case presentation

**Figure 1.**
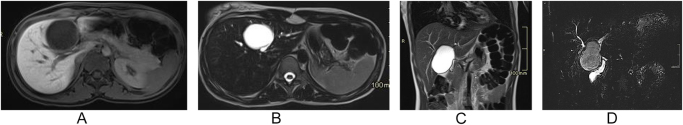
(A–D) MRI liver/MRCP showing a solitary 6.4 cm well-defined, multilocular cystic lesion centered at segment 4b/5 causing bi-lobar compression effect on the hepatic vasculature inflow, along with the biliary drainage. The cyst was not communicating with the biliary system.

**Figure 2.**
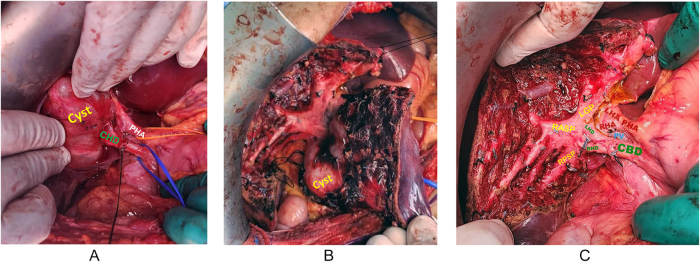
(A–C) Intra-operative pictures. (A) showing the close relation of the cyst with common hepatic duct and the proper hepatic artery. (B) showing the close relation of the cyst with the liver hilar plate. (C) The liver hilar plate after the excision of the cyst. PV, portal vein; CHA, common hepatic artery; PHA, proper hepatic artery; CBD, common bile duct; CHD, common hepatic duct; RHD, right hepatic duct; LHD, left hepatic duct; RPV, right portal vein; LPV, left portal vein; RASP, right anterior sector pedicle; RPSP, right posterior sector pedicle; LGP, left Glissonean pedicle.

**Figure 3.**
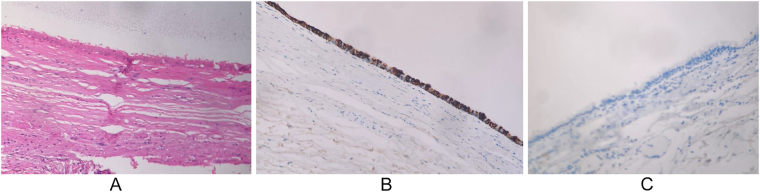
(A–C) Histopathological examination of the excised liver lesion. (A) The lesion’s wall shows pseudostratified ciliated columnar epithelium supported by underlying connective tissue, a smooth muscle layer, and an outer fibrous capsule, evident in hematoxylin and eosin stained. (B) Immunohistochemical staining with Cytokeratin 7 shows diffuse cyst wall uptake. (C) Immunohistochemical staining with Cytokeratin 20 shows no cyst wall uptake.

We report the case of a 19-year-old female referred to our center for further workup after an incidental finding of a liver lesion encountered during a laparoscopic appendectomy for acute noncomplicated appendicitis. The patient had an unremarkable history and physical examination, and there was no history to suggestive of hydatid disease. The blood test results were unremarkable, except for an elevated carbohydrate antigen 19-9 (CA 19-9) level of 109 U/ml (normal range, 0–37 U/ml). In addition, the serological test for hydatid disease was negative. The patient underwent abdominal computed tomography (CT) and liver magnetic resonance imaging (MRI) to further characterize the liver lesion (Fig. 1). Both patients presented with a solitary 6.4-cm well-defined, multilocular cystic lesion centered at segment 4b/5, causing a bi-lobar compression effect on the hepatic vasculature inflow and biliary drainage. The cyst did not communicate with the biliary system and had no radiological features suggesting malignancy. The differential diagnoses included biliary cystadenoma, hepatic cyst, cystic biliary hamartoma, and, although less likely, hydatid cyst. Given these findings and the low suspicion of malignancy, we opted for complete cyst excision without radical liver resection. Intraoperatively, the cyst was stretching the gall bladder, and it was found abutting the bi-lobar liver vasculature inflow and the biliary drainage. Therefore, a cholecystectomy was performed, followed by a nonanatomical wedge resection of segment 4/5. At the liver hilum, the cyst was dissected from the hepatic vasculature inflow and biliary system (Fig. 2). Complete cyst excision was achieved, and the major hepatic hilar structures were preserved. The patient had a smooth postoperative course and was discharged home in good medical condition 4 days after surgery. Final histopathology revealed a CHFC without evidence of malignancy or dysplasia (Fig. 3). Upon follow-up in the outpatient clinic, the CA 19.9 level was requested and found to be within the normal range, suggesting that the previous elevation was most likely a transient inflammatory rise. The patient remained clinically well for 2 years after surgery.

## Discussion

CHFC is a rare benign solitary cystic lesion of the liver. A recent thorough analysis of all the reported cases to December 2024 by Sparrelid *et al* estimated that there were more than 223 cases, of which approximately 15 underwent metaplasia (12 squamous and 3 gastric), and 6 had malignant transformation^[1,2,12]^. This analysis included all cases confirmed by microscopic examination and those diagnosed solely based on radiological features, which may have overestimated the true number of cases^[1,2]^. While CHFCs most commonly occur in segment IV of the liver (83% of cases), they may also be found in the right lobe (15%), porta hepatis, gallbladder, common hepatic duct, or falciform ligament^[2,3,10,12]^. This distribution fits their embryological development, since the left hemiliver, especially segment IV, makes up most of the liver mass during early gestation (weeks 4–6)^[13]^.

CHFCs form because of the migration of embryonic foregut cells from the dorsal bud (which gives rise to the future esophagus and trachea) into the ventral bud (which gives rise to the future liver)^[10]^. According to Al Beteddini *et al*, this pattern of migration explains why the lining of the cyst frequently resembles bronchial tissue, with a ciliated pseudostratified columnar epithelium comparable to that of esophageal and bronchial cysts^[14]^.

CHFCs are traditionally composed of four layers: an outer fibrous capsule, a smooth muscle band, a loose connective tissue layer (lamina propria), and an inner lining of ciliated pseudostratified columnar epithelium. Immunohistochemical staining is critical for determining the foregut origin of CHFCs. These cysts are frequently keratin 7 positive and CDX-2 negative. Furthermore, thyroid transcription factor 1 antibody staining is consistently positive, providing additional diagnostic evidence for this condition. Periodic acid-Schiff staining can be used to identify mucinous content in the epithelium^[12]^.

The clinical presentation of CHFCs varies, but most cases in the literature, including the case discussed in this study, were diagnosed incidentally. However, a few reported cases presented with mass effect symptoms (post-prandial abdominal pain, vomiting, and obstructive jaundice)^[12]^. Hepatic and serology panels are the initial laboratory investigations in such cases to rule out other differential diagnoses (including Mycobacterium tuberculosis and parasitic infections, specifically Echinococcus). Both are important differential diagnoses to be considered before proceeding with the diagnosis of CHFC. While different radiological modalities can assist in the diagnosis of CHFCs, radiographic imaging is not sufficient for diagnosis because of the diversity in appearance^[2,15]^. Typically, CHFCs are small (<4 cm), subcapsular, unilocular, and fluid-filled. A hypoechoic cyst that is hyperdense without contrast enhancement on CT imaging is typically found during ultrasound assessment. On the MRI T1-weighted phase, there is considerable variability, but on the T2-weighted phase, CHFCs are almost exclusively hyperintense. A wide range of imaging results includes simple cysts, biliary cystadenoma, pyogenic abscess, amebic abscess, hydatid cyst, intrahepatic pseudocyst, mucinous cystic neoplasm, and cystadenocarcinoma^[3]^. Lastly, differential diagnoses that may present with similar radiological findings include epidermoid/endodermal cysts, Echinococcus cysts, pyogenic abscesses, cystadenocarcinomas, cystadenomas, choledochal cysts, mesenchymal hamartomas, and hypovascular solid tumors (Table 2) ^[16]^.

**Table 2:** Characteristic MRI findings in the differential Diagnosis of Cystic Liver Lesions

Lesion	T1 Signal	T2 Signal	Contrast Enhancement	Key Distinguishing MRI Features
Ciliated hepatic foregut cyst (CHFC)^[6]^	Variable: hypo-, iso-, or hyper-intense depending on mucin/protein content	Markedly hyperintense	None	Usually small (<4 cm), solitary, subcapsular in segment IV; unilocular, thin-walled; may show fluid–fluid levels
Simple hepatic cyst^[17]^	Hypointense	Homogeneously hyperintense	None	Thin-walled, anechoic, no septations or fluid–fluid levels
Hydatid cyst^[17]^	Variable; may be hypointense with internal membranes	Hyperintense with internal septations/daughter cysts	Peripheral rim enhancement possible	Multiloculated, daughter cysts, calcified rim possible
Mucinous cystic neoplasm (MCN)^[18]^	Hyperintense if mucinous (fluid filled)	Hypointense septa/calcification or hemorrhagic	Enhancing thick wall/septa	Multiloculated, septated, mural nodules possible; often in middle-aged women
Biliary cystadenoma^[19]^	Variable; often hyperintense if mucinous or hemorrhagic	Hyperintense with septa as hypointense lines	Enhancing thick capsule and septations	Multiloculated cystic mass; may have mural nodules; slow growing
Cystic biliary hamartoma^[20]^	Hypointense to isointense	Hyperintense (small, scattered cysts)	No significant enhancement	Multiple tiny cystic lesions throughout liver; no mass effect; normal intervening liver parenchyma
Cystic metastasis^[4]^	Variable; may be hyperintense (melanin, hemorrhage)	Often less hyperintense, heterogeneous	Mural/septal enhancement	Nodularity or solid components; clinical history crucial
Epidermoid/dermoid cyst^[21]^	–	Hyperintense	None unless complicated	Fat suppression reveals fatty components
Caroli disease^[22]^	Hypointense	Hyperintense tubular structures	No enhancement of cystic portions	“Central dot sign,” continuity with biliary tree on MRCP

Although the majority of CHFCs remain benign, approximately 3% undergo malignant transformation, developing into squamous cell carcinoma. A review of the literature by Sparrelid *et al* revealed 223 cases of CHFCs, with an estimated incidence of malignancy of 2.7% (6/223 cases); 5.4% of the cases resembled squamous metaplasia (12/223), whose progression most likely follows a metaplasia–dysplasia–carcinoma sequence^[1]^. According to the malignant transformed cases reported in the literature, the mean average size of CHFC with malignant transformation is 10 cm, which is considered a large cyst. Furthermore, due to the risk of malignancy, it has always been advised to resect these lesions whenever they are suspected during a preoperative workup, especially when they are large and found to have focal wall abnormalities or thick septations on imaging^[1,4]^. However, the presence of all the aforementioned features for malignant transformation does not rule out the possibility of malignant transformation in the absence of these features. Therefore, a potentially malignant CHFC should be resected whenever the size is large, increasing in size, and in young patients due to the risk of malignant transformation. Leaning toward the surgical intervention in the context of malignant transformation is preferred in the literature, but there is no alternative plan in terms of surveillance protocols due to insufficient evidence.

In the context of tumor markers, Muraoka *et al* observed in a six-case series that serum CA 19-9 concentrations may be elevated in a subset of patients with CHFC, although the majority of cases demonstrated values within the reference range. These findings reinforce the rationale for complete surgical excision, even in radiologically or clinically benign-appearing lesions, to mitigate the potential – albeit rare – risk of malignant transformation. Correspondingly, Yoon *et al* reported a histologically benign CHFC exhibiting markedly elevated intra-cystic concentrations of CA 19-9 and CEA, thereby underscoring that such elevations are not pathognomonic for malignancy but may still warrant resection to establish a definitive diagnosis and ensure appropriate management^[4,23]^. Elevated serum CA 19-9 is not only limited to malignant causes (e.g., pancreatic cancer and cholangiocarcinoma) but can also be elevated in acute benign cases such as Hashimoto thyroiditis, diverticulitis, pancreatitis, obstructive jaundice, and cholangitis^[24]^. In relation to our case, the elevation of CA 19-9 currently cannot be linked to the diagnosis of CHFC or its malignant transformation due to insufficient evidence. Subsequently, the elevation of serum CA 19-9, which was encountered in our case, could be justified due to the patient’s acute illness of having appendicitis during the work-up timeframe and the noted compression of the bi-lobular hepatic vasculature and biliary drainage by the CHFC.

Complete surgical excision is typically advised because of the possibility of metaplasia and malignant transformation^[12]^. Most experts contend that surgery is better than long-term surveillance because of the possibility of cancer development, although some recommend monitoring small, asymptomatic cysts^[4]^. Additionally, the risk increases with cyst size, and all documented malignant instances were ≥7 cm. We think this raises the question of whether active surveillance, as opposed to surgery, should be recommended for asymptomatic smaller lesions (<7 cm) with reassuring or suggestive radiological features of CHFCs. However, another review suggested that in cases where fine-needle aspiration or core biopsy confirms the diagnosis of a CHFC, excision of CHFCs ≥3 cm is recommended because of the favorable correlation between size and squamous transformation and the poor prognosis of malignant transformation. Asymptomatic cysts <3 cm should be resected if imaging reveals abnormalities such as wall irregularities or calcifications^[2]^. In the absence of suspicious imaging findings, serial follow-up is required to monitor the growth or changes in the appearance of the cyst; symptomatic cysts require resection regardless of size. Further, sclerotherapy and aspiration are not the best options because they frequently result in recurrence and do not eliminate the risk of cancer^[12]^.

When feasible, laparoscopic resection is the recommended technique because it has few side effects and requires little hospitalization (median: 2 days)^[10]^. Open surgery may be necessary for large lesions or deeply seated cysts^[4]^. Laparoscopic surgery has been documented for certain cases; however, because of its close relationship to the liver hilum, open surgery was considered a safer and more practical alternative for the reported patient. Moreover, there have been no documented recurrences following total excision; therefore, enucleation or wedge resection is typically adequate^[12]^. Postoperative follow-up is usually short because there is no documented recurrence following complete removal^[4]^. There is no established surveillance protocol for untreated cysts; however, to track growth or malignant changes, regular imaging may be required^[12,25]^.

## Conclusion

CHFCs are uncommon benign liver cysts with a 3% risk of malignancy. While most cysts are asymptomatic, young patients, large cysts (≥10 cm), or those with worrisome features should be surgically removed due to the risk of malignant transformation. Laparoscopic excision is favored because it is associated with reduced morbidity and no recurrence. Asymptomatic cysts <3 cm with no atypical features can be monitored with serial imaging for growth or changes in their appearance. This example emphasizes the need to consider CHFC in hepatic cystic lesions and the requirement for complete excision due to the risk of malignancy. Due to the limited number of reported cases, this case is noteworthy in raising the index of suspicion for the younger age group of patients who are at risk of malignant transformation. In addition, the preoperative elevation of CA 19-9 and its value in the diagnostic process of CHFC and the technically challenging hilar dissection required for complete excision. This case highlights the importance of considering CHFC in the differential diagnosis of cystic liver lesions, advocating surgical excision in young patients diagnosed with large cysts and worrisome features. Careful planning of surgical intervention is required when lesions abut the major hepatic vasculature and biliary structures. This report adds to the limited literature by illustrating the practicality of the diagnostic process and surgical considerations in terms of age group and emphasizes the need for further research regarding CHFCs management guidelines and possible surveillance protocols.

## Data Availability

It is a case report, data sharing is not applicable to this article.

## Research registration unique identifying number (UIN)

None. It is a case report no research registration required.
